# Profile of miR-23 Expression and Possible Role in Regulation of Glutamic Acid Decarboxylase during Postnatal Retinal Development

**DOI:** 10.3390/ijms22137078

**Published:** 2021-06-30

**Authors:** Etelka Pöstyéni, Andrea Kovács-Valasek, Péter Urbán, Lilla Czuni, György Sétáló, Csaba Fekete, Robert Gabriel

**Affiliations:** 1 Department of Experimental Zoology and Neurobiology, University of Pécs, 7624 Pécs, Hungary; etelka91@gamma.ttk.pte.hu; 2 János Szentágothai Research Centre, 7624 Pécs, Hungary; urpe.89@gmail.com (P.U.); czuni.lilla@gmail.com (L.C.); fekete@gamma.ttk.pte.hu (C.F.); 3 Department of Medical Biology, Medical School, University of Pécs, 7624 Pécs, Hungary; gyorgy.setalo.jr@aok.pte.hu

**Keywords:** miR-23, retina, *GAD1*, glutamic acid decarboxylase 67, in situ hybridization, next-generation sequencing

## Abstract

As neurotransmitter, GABA is fundamental for physiological processes in the developing retina. Its synthesis enzymes are present during retinal development, although the molecular regulatory mechanisms behind the changes in expression are not entirely understood. In this study, we revealed the expression patterns of glutamic acid decarboxylase 67(GAD67) and its coding gene (GAD1) and its potential miRNA-dependent regulation during the first three postnatal weeks in rat retina. To gain insight into the molecular mechanisms, miRNA-sequencing supported by RT-qPCR and in situ hybridization were carried out. GAD1 expression shows an increasing tendency, peaking at P15. From the in silico-predicted GAD1 targeting miRNAs, only miR-23 showed similar expression patterns, which is a known regulator of GAD1 expression. For further investigation, we made an in situ hybridization investigation where both GAD67 and miR-23 also showed lower expression before P7, with the intensity of expression gradually increasing until P21. Horizontal cells at P7, amacrine cells at P15 and P21, and some cells in the ganglion cell layer at several time points were double labelled with miR-23 and GAD67. Our results highlight the complexity of these regulatory networks and the possible role of miR-23 in the regulation of GABA synthesizing enzyme expression during postnatal retina development.

## 1. Introduction

GABA (gamma-aminobutyric acid) was discovered in the vertebrate brain in 1950, and later its function as an inhibitory neurotransmitter was proved in the central nervous system (CNS) [1,2,3]. It has an indispensable predominant role in the developing mammalian CNS, as it is simultaneously involved in neuronal survival, migration, synaptogenesis and differentiation [4,5,6,7,8,9,10,11].

GABA is synthetized from glutamate by two different isoforms of the glutamic acid decarboxylase (GAD) enzyme, which are distinguished by their molecular weights: GAD65 (~65 kDa) and GAD67 (~67 kDa) [12,13]. These isoforms are coded by two distinct genes (GAD67—*GAD1* gene, GAD65—*GAD2* gene) and their expression patterns are cell type-dependent. The GAD65 isoform is usually localized in synaptic terminals, while GAD67 occurs throughout the cell, including dendrites and the soma [14,15]. Their differential participation in the GABA synthesis in the CNS has been the focus of numerous studies. In knockout experiments, GAD65 knockout (GAD65−/−) mice showed normal GABA levels in the brain, while in another study, the GAD67 heterozygote (GAD67−/+) mice showed significantly lower levels of GABA, although their GAD65 level was normal. These results indicate that GAD67 is essential for GABA synthesis in the brain [16,17].

In the retina, many amacrine and a few ganglion cells are GABA-containing neurons, and this neurotransmitter is also present in the horizontal cells of the vertebrate retina [18] (e.g., cat [19], rabbit [20], monkey [21]). In the horizontal cells of the adult rat and mouse retina, GABA and GAD immunoreactivity is absent, although during the postnatal development it is transiently expressed [22,23]. In the developing rat retina, amacrine cells show increasing GABA immunoreactivity until adulthood, while in horizontal cells, strong GABA labelling occurs until just P15 [23]. In a quantitative study, 99% of amacrine cells co-localized GABA and GAD67-GFP in GAD67-GFP knock-in mice. They found co-localization of GAD67-GFP and GABA in the ganglion cell layer (GCL) and in the inner nuclear layer (INL) of the retina; 25.4% of the displaced amacrine cells and 21.3% of the amacrine cells in the INL were GABA positive [24]. GAD67 expression could be observed from birth, while GAD65 expression arose at the end of the first postnatal week in the rat retina. The distribution of these two enzymes became similar after the fourth postnatal week, but horizontal cells remained just GAD67 positive. GAD67 expression was low at P1 and P4, and the immunoreactivity increased later in the GCL and in the INL in P8 rat retinas. In the GCL, it reached its maximum level at P21 where 38% of the cells showed GAD67 immunoreactivity and decreased after. Meanwhile, in the INL, the expression increased until it reached the values representative of adulthood [25]. GAD67 expression was first detected in horizontal and amacrine cells at P3 but this changed at P10, when the GAD67 expression was reduced in horizontal cells and remained unchanging only in amacrine cells. Otherwise, the expression of the enzyme was increased at P21 in amacrine cells and down-regulated around P14 in horizontal cells [26]. GAD67 immunoreactivity occurred in the amacrine, ganglion and horizontal cells at the end of the 1st postnatal week; after this, the proportion of GAD67 positive horizontal cells increased until the opening of the eye lid. This immunoreactivity disappeared in most horizontal cells during the 3rd week and its appearance became similar to that of the adults just at the end of the 4th postnatal week [27].

Although the expression of GAD67 has already been widely analysed during the developmental process of the retina [25,26,27], the underlying molecular mechanisms involved in the transcriptional regulation during the postnatal development remain unknown. Our approach is innovative in the sense that to our knowledge no one has ever used three methodologies to study a single microRNA (miRNA) in the regulation of transmitter synthesis enzyme. We strongly believe it is important to study these mechanisms from different angles (sequence data, quantitative aspects, tissue distribution). This way, all aspects of the spatiotemporal expression pattern can be followed in substantial detail.

MiRNAs have a tissue-specific expression manner and contribute to the regulation of many physiological processes in the CNS, including development and maturation [28,29,30]. During retinal development, miRNAs are important key regulators in a variety of cellular processes and have a unique spatiotemporal expression pattern [31,32,33,34]. We employed different publicly available miRNA–target interactions platforms to determinate experimentally validated miRNAs, which are potentially *GAD1* specific (miRTarBase, MiRanda (miR-23), Miranda (miR-216a, miR-216b, miR-493, miR-495, miR-542)). This study aims to gain a deeper understanding of the molecular regulatory networks that contribute to the final expression pattern formation of the major GABA synthetizing enzyme GAD67 during the first three weeks of the postnatal retinal development.

## 2. Results

**Figure 1:**
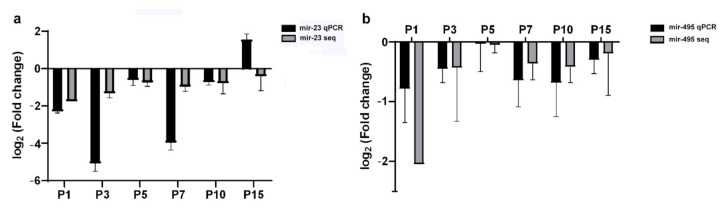
The expression alteration of miR-23 (**a**) and miR-495 (**b**) revealed by RNA-sequencing (grey) and RT-qPCR measurements (black). Fold changes are represented as log2 Fold change compared to P21 (Table S1).

**Figure 2:**
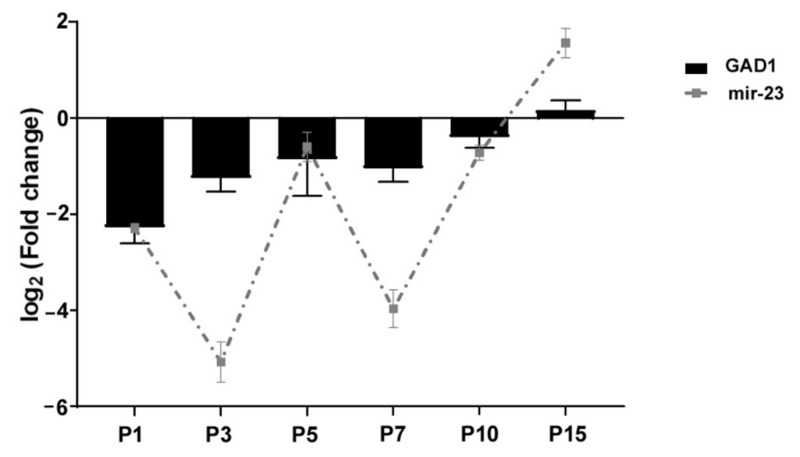
Relative expression levels of miR-23 (dashed line) and its target gene GAD1 (black) determined by RT-qPCR. Target gene expression level was normalized against glyceraldehyde-3-phosphate dehydrogenase (GAPDH) as an internal control gene. The change in miR-23 expression was calculated with U6snRNA as a reference gene for normalization. Expression was compared with P21 (Tables S1 and S2).

We performed a miRNA-Seq study to obtain a deeper insight into the background of gene expression regulation at the miRNA level of retinal cell development (GSE159168). From this study, we obtained postnatal expression patterns of GAD1 related miRNAs (miR-216a, miR-216b, miR-493; miR-23, miR-542, miR-495; Figure S1). Subsequently, we revealed these miRNAs and GAD1 mRNA postnatal expression patterns by RT-qPCR measurements. Based on their expression pattern we could classify these miRNAs into two groups: the first group with similar patterns to GAD1 expression (both miR-23 and miR-495 are lower at most examined time points than at P21) (Figure 1 and Figure 2; Table S2) and the second group with non-matching expression patterns (miR-542, miR-216a, miR-216b, miR-493; Figure S2, Table S1).

**Figure 3:**
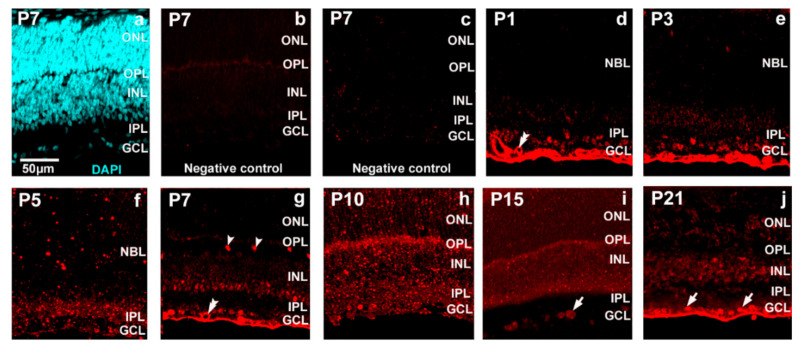
The cellular localization of miR-23 in the Wistar rat retina at the first 3 postnatal weeks by in situ hybridization ((**d**) P1; (**e**) P3; (**f**) P5; (**g**) P7; (**h**) P10; (**i**) P15; (**j**) P21). The retina has been counterstained with DAPI (**a**) and controls (**b**) without anti-miR-23, (**c**) without anti-DIG-HRP are presented. Arrowheads indicate horizontal cells, double arrowheads indicate capillary and cells in the GCL are indicated with arrows. The scale bar represents 50 µm. GCL—ganglion cell layer; IPL—inner plexiform layer; NBL—neuroblast layer; INL—inner nuclear layer; OPL—outer plexiform layer; ONL—outer nuclear layer.

**Figure 4:**
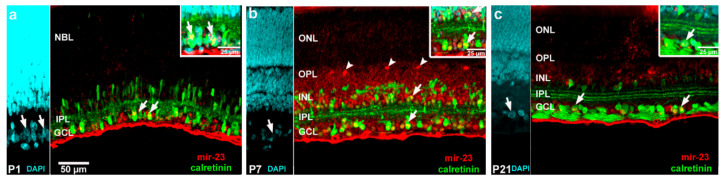
Representative images of miR-23 (red: TSA Cy 3) co-detection with calretinin (green: Alexa Fluor-488) at the indicated postnatal days (miR-23 with calretinin: (**a**) P1; (**b**) P7; (**c**) P21). Inserted images demonstrate the overlap of ISH-dual IHC. Arrows indicated co-localizations in cells in the GCL and the INL, while non-colocalized cells (horizontal cells) were demonstrated by arrowheads. Scale bar in retina sections: 50 µm (both DAPI and IHC), in inserts: 25 µm. GCL—ganglion cell layer; IPL—inner plexiform layer; NBL—neuro blast layer; INL—inner nuclear layer; OPL—outer plexiform layer; ONL—outer nuclear layer.

In our previous methodical study, we also observed a time-dependent expression of miR-23 in the rat retina [35], while an earlier microarray study concluded in the CNS has already described its regulatory role on GAD1 gene expression [36]. Consequently, we choose miR-23 for further analysis by in situ hybridization (ISH) in our investigation (Figure 3 and Figure 4).

**Figure 5:**
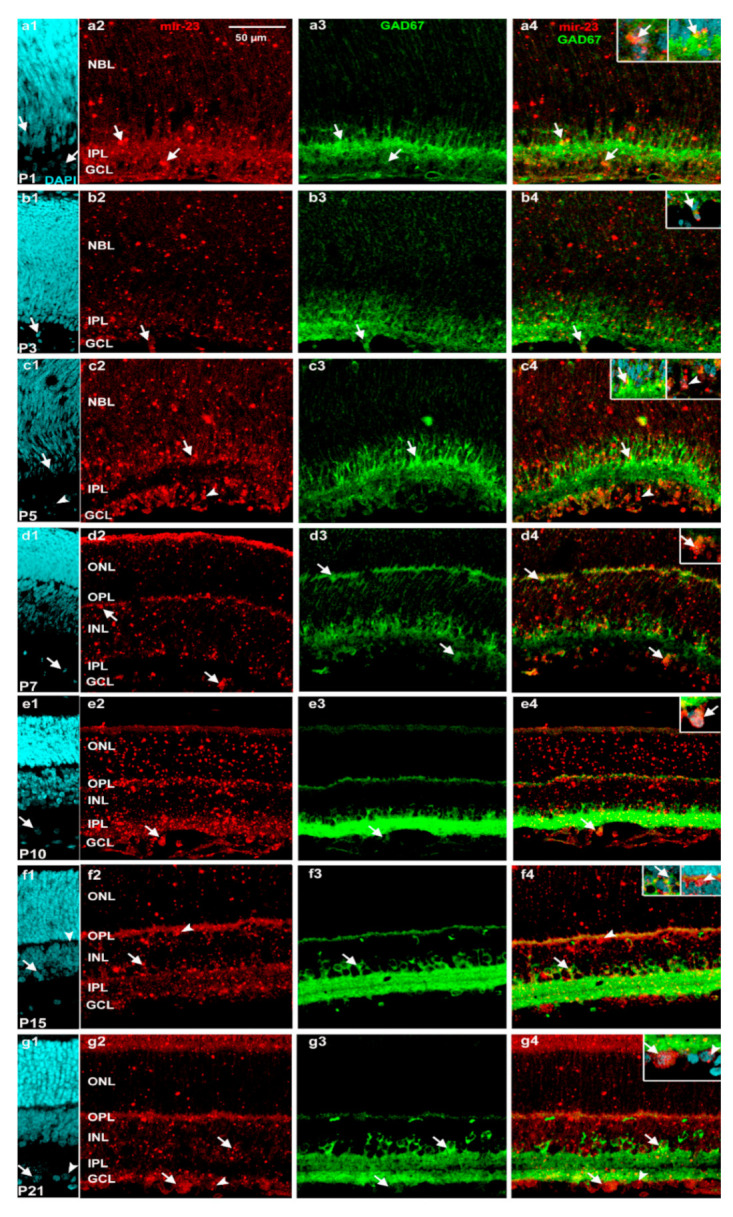
Characterization of miR-23 (red: TSA Cy3) expressing cells (in situ hybridization) and GAD67 (green: Alexa Fluor-488) by immunolabeling in postnatal developing retina sections of indicated ages ((**a1**–**a4**) P1; (**b1**–**b4**) P3; (**c1**–**c4**) P5; (**d1**–**d4**) P7; (**e1**–**e4**) P10; (**f1**–**f4**) P15; (**g1**–**g4**) P21). The first column demonstrates the DAPI staining, the second column miR-23 and third column represent GAD67 staining. The fourth column demonstrates the merged images of ISH–IHC staining. Arrows indicate colocalization, and non-colocalized cells were demonstrated by arrowheads. Scale bar—50 µm on panel a2. NBL—neuroblastic layer, GCL—ganglion cell layer, IPL—inner plexiform layer, INL—inner nuclear layer, OPL—outer plexiform layer, ONL—outer nuclear layer.

Retinal tissue has been routinely counterstained with 4′,6-diamidino-2-phenylindole (DAPI) (Figure 3a). Controls (Figure 3b—without anti-miR-23, Figure 3c—without anti-digoxigenin-horseradish peroxidise (anti-DIG-HRP)) did not show any staining. When capillaries occurred in the retina sections, they showed miR-23 positivity (Figure 3d,g). The presence of miR-23 was low before P7 (Figure 3d–f), where horizontal cells showed remarkable hybridization-related tyramide signal amplification (TSA) fluorescence (Figure 3g). Strong labelling occurs in the GCL (Figure 3i,j). To confirm that miR-23 signals belong to different cell types, co-detection of miR-23 and calretinin was executed (Figure 4). Mir-23 showed co-labelling in some cells in the GCL (Figure 4a–c) and the horizontal cells shows miR-23 positivity at P7 (Figure 4b). Immunofluorescence staining indicated that GAD67 is expressed in the retina during development, especially in the inner plexiform layer (IPL) and the GCL, and to a lesser extent, in the outer plexiform layer (OPL) and INL (Figure 5).

GAD67 expression was less prominent before P7 than later and some cells in the GCL appeared double-labelled (33% of the GAD67 positive cells were miR-23 positive too in the INL and 90,5% in the GCL; Figure 5(a1–c4) and Table S3), while at P7 both markers could be detected in horizontal cells (Figure 5(d4)). After P7, the GAD67 staining intensity gradually increased and showed elevated levels both at P10 (Figure 5(e1–e4)) and P15 (Figure 5(f1–f4)), then decreased by P21 in the examined retinas. In the last two time points, at P15 (Figure 5(f4)) and P21 (Figure 5(g4)), only a few amacrine cells (12%) were double labelled in the INL. At P10 (Figure 5(e4)) and P21 (Figure 5(g4)), we found double labelled presumed ganglion cells in the GCL (75%) (Table S3).

## 3. Discussion

Our findings support the results of previous investigations presenting the changing expression pattern of the major GABA synthesis enzyme GAD67 during postnatal development in the retina, although not all the aspects of the underlying influential molecular mechanisms could be fully revealed. MiRNAs are essential participants of the molecular regulatory processes of gene expression; moreover their critical control function in biological processes of retina has also been confirmed [31,33,37,38,39]. Acquiring data with three different but related methodologies allows us to draw rational conclusions on the degree of involvement of miR-23 in the fine regulation of GAD67. In order to obtain deeper insights into the GAD67 expression regulation in the retina (i) we have characterized the time-dependent expression alteration of *GAD1* and (ii) different miRNAs which potentially target this gene during the first three postnatal weeks, and (iii) the whole process has been followed by in situ hybridization in the retinal tissue.

Of the six miRNAs potentially targeting *GAD1* gene, the role of miR-23 has clearly stood out in the retina (including the pigment epithelium) [32,33,38,40], although none of the previous papers included tissue distribution data. At the same time, miR-23 contribution to *GAD1* expression modulation is also supported by a CNS microarray study [36].

Of the different retinal cell types, bipolar cells, Müller glia, some amacrine cells and the majority of rod photoreceptors are generated postnatally [41,42]. During this postnatal period, the time before eye lid opening (P14) is critical in the development because the synaptogenesis in the synaptic layers begins at this time around P10 and reaches its maturity at P21 [43,44].

In our in situ hybridization investigation of both miR-23 and GAD67 showed lower expression until P7 (Figure 3 and Figure 5(a3–c3)) and similarly, the *GAD1* mRNA expression was also lower during this period (Figure 2). When capillaries appeared in retinal sections, miR-23 positivity was remarkably strong (Figure 3d,g) and this is in accord with previous studies which also described the importance of miR-23 in vascularization of the retina by enhancing angiogenesis [45]. At P7 (Figure 3g and Figure 4b) horizontal cells were miR-23 positive and showing co-labelling with GAD67 at the same time (Figure 5(d4)). This co-labelling disappeared in horizontal cell bodies and only the miR-23 labelling remained at P15. Previous studies also mentioned the same disappearance or down-regulation of GAD67 positivity in horizontal cells at the end of the second postnatal week [25,26]. Likewise, GABA expression is also transient in the outer retina of rat during postnatal development and disappears in horizontal cells after P15, while in the inner retinal layers GABA labelling is increasing until adulthood [23]. In previous investigations, miR-23 was described showing high expression both in the brain and the retina [32,33] particularly after P12 [38]. The highest *GAD1* expression occurs at P15, where miR-23 also has the highest value according to qPCR results, compared to the other time points (Figure 1 and Figure 2). Yamasaki and colleagues have also described that GAD67 expression was lower during early development and increased just after P8 in the GCL and in the INL [25]. Moreover, GABA expression also increased after P6 in the ganglion and amacrine cells and remained unchanged until P12 [23]. At P15 and P21 we observed miR-23 positive cells co-labelled with calretinin (Figure 4a–c); moreover, at these time points, there were amacrine cells (Figure 5(f4,g4)) and some cells in the GCL (Figure 5(b4,d4,e4,g4)) co-labelled with GAD67 and miR-23. In a previous study, GAD67 expression was also detected in amacrine cells at the beginning of the postnatal development, followed by an increase before reaching P21 [26]. The presence of GAD67 in the GCL has also been described before, reaching its maximum at P21 [24,25]. The miR-23 occurrence and its possible regulatory role in the developing retina (especially in ganglion cells) have also been revealed in other studies [35,46]. In a previous study with developing rat retina, more GAD67 positive cells in these retinal layers were seen after P8. Their number increased in the INL until adulthood, while in the GCL it reached a maximum at P21 and only later decreased [25].

Development of GABA-containing retinal cells has been investigated in many previous studies although the miRNA regulation of molecular processes in the background remained unknown. Regulator function of miRNAs is vitally important for controlling molecular pathways of developmental processes. Previous microarray and in situ examinations revealed the spatiotemporal occurrence of several miRNAs in the retina and through their combined expression, miRNA-dependent changes have been described during sequential actions of retinal development [31,38,47]. miRNA contribute to the regulation of molecular processes on varying levels: for one, they contribute not only at post-transcriptional level (mRNA degradation [48], repression of translation [49]) where they could target several mRNA, and the same mRNA could be targeted by multiple miRNAs. Furthermore, they can also regulate expression at the level of transcription, where they mediate the activation or inhibition of the target gene transcription [50,51,52,53]. Our results highlight the significance of the transcriptional regulatory role of miR-23 expression through the induction of *GAD1* transcription during postnatal retina development. Our study focusing on this special miRNA regulatory mechanism shed some light on the shaping of the inhibitory network of the retina and stresses the need for continued investigation for a better understanding of the miRNA regulation of this process. In conclusion, these findings underscore the importance of miRNAs regulation at multiple levels of the cellular fate determining molecular networks and the necessity of further investigations about the miRNA involvement in retina developmental processes.

## 4. Materials and Methods

### 4.1. Animal Procedures

The animals were treated according to the regulation of the ARVO Statement for the Use of Animals in Ophthalmic and Vision Research. Experimental protocol was approved by the Ethical Guidelines at the University of Pécs (BAI/35/51-58/2016).

In this study, we used Wistar rats aged 1 (P1) to 21 (P21). Animals were housed under light/dark cycles of 12:12 h, and food and water were available ad libitum. They were anesthetized by inhalation using Forane prior to sacrifice and the harvesting processes were executed at the same hour of the day to avoid circadian variations in miRNA expression. Following euthanasia, the eyes were dissected, and the eyecups were prepared and fixed in 4% paraformaldehyde (PFA, pH 7.4) for 20 min, cryoprotected, embedded in Shandon Cryomatrix, cut at 12 µm in a cryostat, mounted onto Super Frost Ultra Plus slides and stored at −80 °C until use. In other cases, eyes were removed and then retinas were fixed in the above fixative after dissection, washed in ice-cold RNase-free phosphate-buffered saline (PBS) and stored at −80 °C until use.

### 4.2. Nucleic Acid Isolation and Small RNA Sequencing

This methodology was developed for studying other miRNAs and has been previously described [34].

Briefly, small and large RNAs were extracted from the fresh retina tissue, using the NucleoSpin miRNA kit (Macherey–Nagel, Düren, Germany) according to the manufacturer’s instructions. An Agilent Bioanalyzer 2100 (Agilent Technologies, Santa Clara, CA, USA) was utilized to measure the quantity and quality of the RNA. Two Agilent Kits (Agilent Small RNA Kit for microRNAs and the Agilent RNA 6000 Nano Kit for total RNA) were used for this purpose. The miRNAs were measured by the Qubit microRNA Assay Kit (ThermoFisher Scientific, Waltham, MA, USA). For sequencing, three RNA samples (RNA integrity number (RIN) > 7) from retinas of each age group were pooled. In brief, before ligation with sequencing adapters, pooled RNAs were enriched for small RNAs. The ligated RNA sample was reverse transcribed, then purified and size-selected with a magnetic bead-based Cleanup Module, barcodes were assigned (Ion Xpress RNA-Seq Barcode 01–16 Kit). Total RNA-Seq Kit v2 (Thermo Fisher Scientific, Waltham, MA, USA) was used to create libraries. The quality and quantity of the libraries were detected via the High Sensitivity Chip of Agilent Bioanalyzer 2100 (Agilent Technologies, Santa Clara, CA, USA). Diluted libraries were clonally amplified for template preparation by emulsion PCR in the Ion OneTouch 2 system using the Ion PGM Template OT2 200 kit. Templated ISPs were loaded onto an IonTorrent 316 Chip and run on the Ion Personal Genomics Machine (PGM) (ThermoFisher Scientific, Waltham, MA, USA). The Ion PGM system generated raw files which were trimmed, then mapped to the noncoding RNAs from ENSEMBL using the automated pipeline of the Ion Torrent Suite Software. Aligned BAM files were uploaded into a Galaxy web-based platform [54]. Relative abundances of transcripts were estimated applying Cufflinks, then several Cufflinks were merged via Cuffmerge. Finally, to detect significant changes in transcript expression, the Cuffdiff application was used [55]. The data have been deposited in the NCBI’s Gene Expression Omnibus [56] and are accessible through GEO Series accession number GSE159168 (https://www.ncbi.nlm.nih.gov/geo/query/acc.cgi?acc=GSE159168, accessed on 1 December 2021). Analysation and visualization of the datasets were executed in an R Studio Software environment [57]. miRNAs potentially targeting *GAD1* gene were predicted by the miRTarBase (http://mirtarbase.cuhk.edu.cn/php/index.php, accessed on: 25 November 2018) and MiRanda (http://www.microrna.org, accessed on: 18 February 2018).

### 4.3. Reverse Transcription and Real Time PCR Quantitation

A two-step qPCR was carried out for miRNA detection. To generate cDNA, synthesis from 10 ng of total RNA using the TaqMan MicroRNA Assay Kit (ThermoFisher Scientific, Waltham, MA, USA) and qPCR using the StepOne Plus qPCR System (ThermoFisher Scientific, Waltham, MA, USA) was performed. The miRNA-specific TaqMan MicroRNA Assays were applied (miR-23: Assay ID:000399, miR-495: Assay ID:001663, miR-493: Assay ID:001040, miR-542: Assay ID:001284, miR-216a: Assay ID:002220, miR-216b: Assay ID:002326) and U6 (Assay ID:001973) was used as an endogenous control. The relative expression levels of each miRNA were determined using the 2^−ΔΔCt^ method [58] and log2-transformed each microRNA/U6 expression ratios were used for further analysis. 

During mRNA analysis, 1 μg of total RNA was used with the RevertAid Reverse Transcriptase (ThermoFisher Scientific, Waltham, MA, USA) and random hexamer primers to generate cDNA according to the manufacturer’s instructions. The predicted target of microRNAs was amplified using gene-specific TaqMan assays (*GAD1*: Rn00690300-m1). The thermal profile was as follows: 95 °C for 10 min of initial denaturation followed by 40 cycles of 95 °C for 15 s to denature and 60 °C for 60 s to anneal and extend. Relative product quantities were determined using StepOne software and the 2^−ΔΔCt^ analysis method [58] using glyceraldehyde 3-phosphate dehydrogenase (GAPDH; Rn01749022_g1) as an endogenous control; RQ values are presented ± SEM (one-way ANOVA following Tukey’s post hoc analysis; * *p* < 0.05, ** *p* < 0.01, *** *p* < 0.001).

### 4.4. Combined microRNA In Situ Hybridisation and Immunocytochemistry

Slides stored for microanatomical investigations were used to detect miR-23 (probe from Exiqon, Cat#:18119-01, sequence: GGAAATCCCTGGCAATGTGAT) expression patterns by implementing ISH method in retinal tissue sections [35]. Sections were post-fixed for 10 min in 4% paraformaldehyde (PFA), treated with proteinase K (5 mg/mL, Sigma-Aldrich, Budapest, Hungary). Acetylation was performed in acetic anhydride/triethanolamine and pre-hybridized in a hybridization solution for 4 h (50% formamide, 0.3 M sodium-chloride (pH 7), 20 mM Tris-HCl; 5 mM EDTA, 1× Denhardt’s solution, 0.5 mg/mL yeast tRNA, 1× Denhardt’s solution). Tissues were hybridized with 5′ DIG-labelled LNA probe in a hybridization solution overnight at 30 °C below RNA probe Tm. As negative controls, slides incubated without a microRNA probe or without anti-DIG horseradish peroxidase. After hybridization, slides were washed with saline–sodium citrate (SSC) at 65 °C and incubated at room temperature with a blocking solution (30 min, Roche, Budapest, Hungary). A second block in a TNB (Tris–NaCl blocking) buffer (0.1 M Tris-HCl (pH 7.5), 0.15 M NaCl, 0.5% Blocking Reagent (Roche, Budapest, Hungary)) was also applied.

Sections were incubated with a mouse anti-DIG horseradish peroxidase antibody (1:500, Perkin Elmer, Per-Form Hungary Kft., Budapest, Hungary) and with rabbit anti-calretinin (1:1000, Swant, Marly, Switzerland; CR7697). For the target immunostaining, we used mouse anti-GAD67 (1:100, Abcam, Cambridge, UK, ab26116) and we incubated sections in a humidified chamber overnight.

Sections were than washed in TNT (Tris–NaCl–Tween) buffer, the primary antibodies were detected using Alexa Fluor 488 (1:1000, Invitrogen, Carlsbad, CA, USA, A11034). After thorough washing in the TNT buffer (3 × 5 min), in situ hybridization signals were revealed using the TSA system according to the manufacturer’s instructions (1:50, Perkin Elmer, Per-Form Hungary Kft., Budapest, Hungary). After repeated washing in TNT (3 × 5 min) we mounted the sections in Prolong Gold antifade reagent with DAPI before being analysed in a confocal microscope (Olympus IX 81 inverse plat-form—Olympus Fluoview FV-1000 Laser Confocal Scanning Microscope; Olympus, Tokyo, Japan). We have counted the ratio of miR-23, GAD67 positive and co-labelled cells in every group (7–8 microscope frames/groups; Table S3).

## Data Availability

The data presented in this study are available in Supplementary Materials (Figures S1 and S2, Tables S1 and S2).

## Supplementary Materials

The following are available online at https://www.mdpi.com/article/10.3390/ijms22137078/s1, Figure S1: Expression alteration of miRNAs revealed by RNA-sequencing. Fold changes are represented as log2 Fold change compared to P21, Figure S2: Expression alteration of miRNAs revealed by qPCR measurements. The change in the miRNA expression was calculated with U6snRNA as reference gene for normalization. Fold changes are represented as log2 Fold change compared to P21, Table S1: Multiple comparison of miRNAs expression in different time points analysed by ordinary one-way ANOVA with Tukey’s post-hoc test. Data obtained from RT-qPCR and miRNA-sequencing (* *p* < 0.05, ** *p* < 0.01, *** *p* < 0.001, **** *p* < 0.0001). Table S2: Multiple comparison of GAD1 mRNA expression at different time points analysed by ordinary one-way ANOVA with Tukey’s post-hoc test. (* *p* < 0.05, ** *p* < 0.01, *** *p* < 0.001, **** *p* < 0.0001). Table S3: Ratio of GAD67 positive, miR-23 positive and co-labelled cells in every group (a.,) and the ratio of GAD67 positive cells before and after P7 (b.,).
