# Supplementary material for: Profile of miR-23 Expression and Possible Role in Regulation of Glutamic Acid Decarboxylase during Postnatal Retinal Development
Source: Int J Mol Sci. 2021 Jun 30;22(13):7078. doi: 10.3390/ijms22137078 (PMC8268301; doi:10.3390/ijms22137078)
Supplement: Supplementary file 1 [file ijms-22-07078-s001.zip › ijms-1244171-supplementary.pdf]

International Journal of
Molecular Sciences

MDPI

Article

Profile of mir-23 expression and possible role in regulation of glutamic acid decarboxylase during postnatal retinal development

Etelka Pöstyéni [1], Andrea Kovács-Valasek[1*], Péter Urbán[2], Lilla Czuni[2], György Sétáló Jr.[3], Csaba Fekete[2] and Robert Gabriel[1*]

[1] Department of Experimental Zoology and Neurobiology, University of Pécs, 7624 Pécs, Hungary; etelka91@gamma.ttk.pte.hu (E.P.); valasek@gamma.ttk.pte.hu (A.K.-V.); gabriel@ttk.pte.hu (R.G.)
[2] János Szentágothai Research Centre, Pécs, 7624, Hungary; urpe.89@gmail.com (P.U.); czuni.lilla@gmail.com (L.C.), fekete@gamma.ttk.pte (C.F.)
[3] Department of Medical Biology, Medical School, University of Pécs, 7624 Pécs, Hungary; gyor-gy.setalo.jr@aok.pte.hu (G.S.J.)
* Correspondence: gabriel@ttk.pte.hu, valasek@gamma.ttk.pte.hu

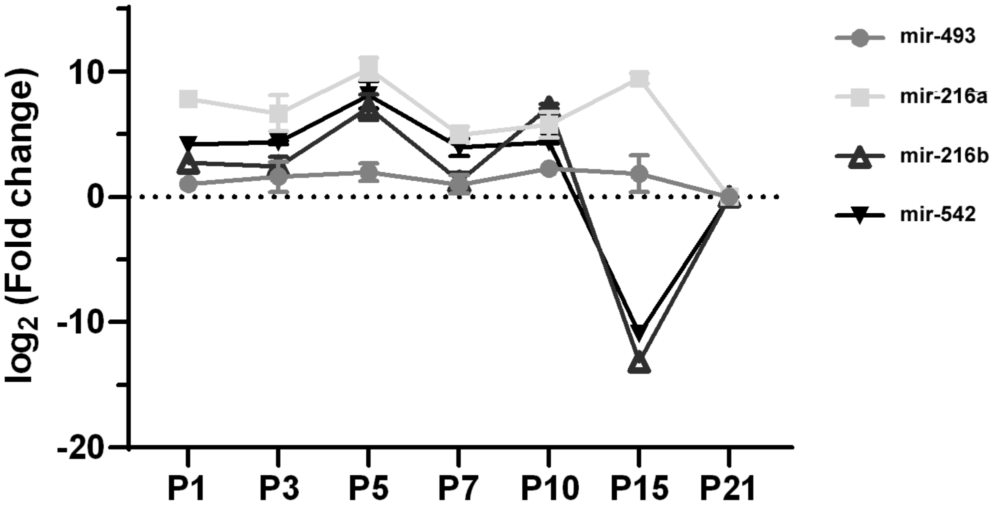

Figure S1. Expression alteration of miRNAs revealed by RNA-sequencing. Fold changes are represented as log2 Fold change compared to P21.

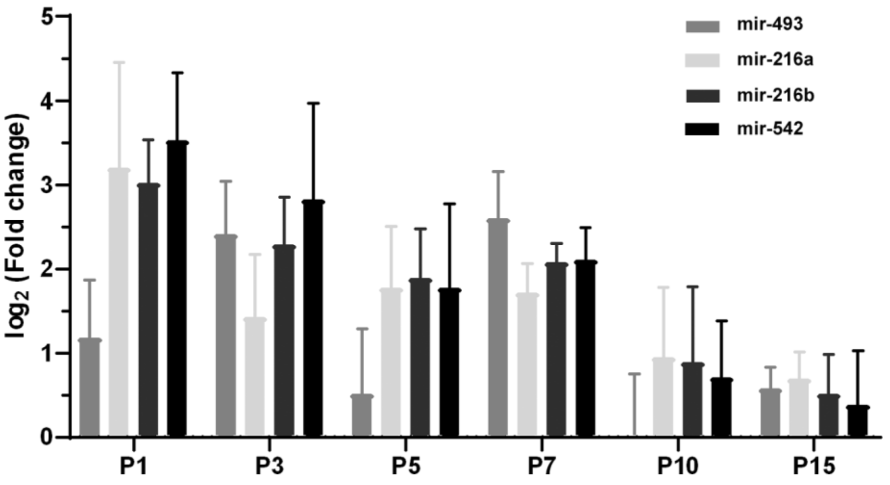

Figure S2. Expression alteration of miRNAs revealed by qPCR measurements. The change in the miRNA expression was calculated with U6snRNA as reference gene for normalization. Fold changes are represented as log2 Fold change compared to P21.

Table S1. Multiple comparison of miRNAs expression in different time points analyzed by ordinary one-way ANOVA with Tukey's post-hoc test. Data obtained from RT-qPCR and miRNA-sequencing (* p<0.05, ** p<0.01, *** p<0.001, **** p<0.0001).

		P1	P3	P5	P7	P10	P15	P21
mir-25 RT-qP	P1	-	****<br><0.0001	****<br><0.0001	***<br>0.0001	***<br>0.0004	****<br><0.0001	****<br><0.0001

P3		-	**** <0.0001	* 0.0298	**** <0.0001	**** <0.0001	**** <0.0001
P5			-	**** <0.0001	ns	**** <0.0001	ns
P7				-	**** <0.0001	**** <0.0001	**** <0.0001
P10					-	**** <0.0001	ns
P15						-	*** 0.0004
P21							-

mir-23 miRNA-Seq

	P1	P3	P5	P7	P10	P15	P21
P1	-	ns	* 0.0162	ns	ns	** 0.0017	**** <0.0001
P3		-	ns	ns	ns	ns	*** 0.0002
P5			-	ns	ns	ns	ns
P7				-	ns	ns	* 0.0262
P10					-	ns	ns
P15						-	ns
P21							-

mir-216a RT-qPCR

	P1	P3	P5	P7	P10	P15	P21
P1	-	** 0.0022	* 0.0257	* 0.0169	**** <0.0001	**** <0.0001	**** <0.0001
P3		-	ns	ns	ns	ns	* 0.0255
P5			-	ns	ns	ns	** 0.0021
P7				-	ns	ns	** 0.0035
P10					-	ns	ns
P15						-	ns

**P21**							-

mir-216a miRNA-Seq

	P1	P3	P5	P7	P10	P15	P21
**P1**	-	ns	**** <0.0001	**** <0.0001	*** 0.0002	** 0.007	**** <0.0001
**P3**		-	**** <0.0001	** 0.0037	ns	**** <0.0001	**** <0.0001
**P5**			-	**** <0.0001	**** <0.0001	ns	**** <0.0001
**P7**				-	ns	**** <0.0001	**** <0.0001
**P10**					-	**** <0.0001	**** <0.0001
**P15**						-	**** <0.0001
**P21**							-

mir-216b RT-qPCR

	P1	P3	P5	P7	P10	P15	P21
**P1**	-	ns	** 0.0089	ns	**** <0.0001	**** <0.0001	**** <0.0001
**P3**		-	ns	ns	*** 0.0005	**** <0.0001	**** <0.0001
**P5**			-	ns	* 0.0314	*** 0.0007	**** <0.0001
**P7**				-	** 0.005	**** <0.0001	**** <0.0001
**P10**					-	ns	ns
**P15**						-	ns
**P21**							-

mir-216b miRNA-Seq

	P1	P3	P5	P7	P10	P15	P21
**P1**	-	ns	**** <0.0001	**** <0.0001	**** <0.0001	**** <0.0001	**** <0.0001
**P3**		-	**** <0.0001	** 0.0011	**** <0.0001	**** <0.0001	**** <0.0001

**P5**			-	**** <0.0001	ns	**** <0.0001	**** <0.0001
**P7**				-	**** <0.0001	**** <0.0001	*** 0.0009
**P10**					-	**** <0.0001	**** <0.0001
**P15**						-	**** <0.0001
**P21**							-

mir-495 RT-qPCR

	**P1**	**P3**	**P5**	**P7**	**P10**	**P15**	**P21**
**P1**	-	ns	ns	ns	ns	ns	**** <0.0001
**P3**		-	ns	ns	ns	ns	**** <0.0001
**P5**			-	ns	ns	ns	**** <0.0001
**P7**				-	ns	ns	**** <0.0001
**P10**					-	ns	**** <0.0001
**P15**						-	**** <0.0001
**P21**							-

mir-495 miRNA-Seq

	**P1**	**P3**	**P5**	**P7**	**P10**	**P15**	**P21**
**P1**	-	** 0.0079	*** 0.0004	** 0.0048	** 0.0074	** 0.0014	*** 0.0003
**P3**		-	ns	ns	ns	ns	ns
**P5**			-	ns	ns	ns	ns
**P7**				-	ns	ns	ns
**P10**					-	ns	ns
**P15**						-	ns

**P21**							-

mir-542 RT-qPCR

	**P1**	**P3**	**P5**	**P7**	**P10**	**P15**	**P21**
**P1**	-	ns	*** 0.0002	** 0.0047	**** <0.0001	**** <0.0001	**** <0.0001
**P3**		-	ns	ns	**** <0.0001	**** <0.0001	**** <0.0001
**P5**			-	ns	ns	** 0.0065	*** 0.0002
**P7**				-	** 0.006	*** 0.0003	**** <0.0001
**P10**					-	ns	ns
**P15**						-	ns
**P21**							-

mir-542 miRNA-Seq

	**P1**	**P3**	**P5**	**P7**	**P10**	**P15**	**P21**
P1	-	ns	**** <0.0001	ns	ns	**** <0.0001	**** <0.0001
P3		-	**** <0.0001	ns	ns	**** <0.0001	**** <0.0001
P5			-	**** <0.0001	**** <0.0001	**** <0.0001	**** <0.0001
P7				-	ns	**** <0.0001	**** <0.0001
P10					-	**** <0.0001	**** <0.0001
P15						-	**** <0.0001
P21							-

mir-493 RT-qPCR

	**P1**	**P3**	**P5**	**P7**	**P10**	**P15**	**P21**
**P1**	-	ns	ns	* 0.0198	ns	ns	ns

**P3**		-	*** 0.0005	ns	**** <0.0001	*** 0.0008	****
**P5**			-	**** <0.0001	ns	ns	ns
**P7**				-	**** <0.0001	*** 0.0002	**** <0.0001
**P10**					-	ns	ns
**P15**						-	ns
**P21**							-

mir-493 miRNA-Seq

	P1	P3	P5	P7	P10	P15	P21
**P1**	-	ns	ns	ns	ns	ns	ns
**P3**		-	ns	ns	ns	ns	** 0.0058
**P5**			-	ns	ns	ns	*** 0.0003
**P7**				-	ns	ns	ns
**P10**					-	ns	**** <0.0001
**P15**						-	*** 0.0009
**P21**							-

Table S2. Multiple comparison of GAD1 mRNA expression at different time points analyzed by ordinary one-way ANOVA with Tukey's post-hoc test. (* p<0.05, ** p<0.01, *** p<0.001, **** p<0.0001).

	P1	P3	P5	P7	P10	P15	P21
**P1**	-	*** 0.0006	**** <0.0001	**** <0.0001	**** <0.0001	**** <0.0001	**** <0.0001
**P3**		-	ns	ns	** 0.0048	**** <0.0001	**** <0.0001
**P5**			-	ns	ns	*** 0.0006	** 0.0055
**P7**				-	ns	**** <0.0001	*** 0.0004

**P10**					-	ns	ns
**P15**						-	ns
**P21**							-

Table 3. Ratio of GAD67 positive, mir-23 positive and colabelled cells in every group (a.,) and the ratio of GAD67 positive cells before and after P7 (b.,).

a.,

	INL			GCL		
	mir-23 positive cell	GAD67 positive cell	colabelled	mir-23 positive cell	GAD67 positive cell	colabelled
P1	0%	50%	50%	30%	9%	61%
P3	38%	63	0%	33%	17%	50%
P5	10%	60%	30%	33%	0%	67%
P7	0%	62%	38%	34%	0%	66%
P10	0%	71%	29%	57%	0%	43%
P15	0%	96%	4%	40%	60%	0%
P21	3%	84%	13%	86%	0%	14%

b.,

	INL		GCL	
	GAD67 positive cells	colabelled	GAD67 positive cells	colabelled
Before P7	67%	33%	9%	91%
After P7	88%	12%	25%	75%
